# Plasmon-Induced Transparency Based on Triple Arc-Ring Resonators

**DOI:** 10.3390/ma11060964

**Published:** 2018-06-06

**Authors:** Guang-Xi Dong, Qin Xie, Qi Zhang, Ben-Xin Wang, Wei-Qing Huang

**Affiliations:** 1 School of Science, Jiangnan University, Wuxi 214122, China; 1130515112@vip.jiangnan.edu.cn (G.-X.D.); 1130515207@vip.jiangnan.edu.cn (Q.X.); hxzhujnu@163.com (Q.Z.); 2 School of Physics and Electronics, Hunan University, Changsha 410082, China

**Keywords:** terahertz, metamaterials, plasmon-induced transparency

## Abstract

This paper presents a plasmon-induced transparency (PIT) using an easy-fabricating metamaterial composed of three pieces of metallic arc-rings on top of a dielectric substrate. The transmission of the transparent peak of 1.32 THz reaches approximately 93%. The utilization of the coupled Lorentzian oscillator model and the distribution of electromagnetic fields together explain the cause of the transparent peak. The simulation results further demonstrate that the bandwidth of the transmission peak can be narrowed by changing the sizes of the arc-rings. Moreover, an on/off effect based on the transparent peak is discussed by introducing photosensitive silicon into the air gaps of the suggested metamaterial structure.

## 1. Introduction

Electromagnetic metamaterials [1,2,3], or artificial structures, can be tailored and controlled to realize some expected functions, such as negative refraction [4], cloaking [5], and so on. Besides, metamaterials can also be designed for use in a specific device, like absorbers [6,7], filters [8,9,10], switches [11], and so on. Among them are electromagnetic-induced transparency (EIT)-based devices [12,13], which have been explored for uses in slowing light [14] and as planar sensors [15]. The EIT is a special phenomenon which originates from the coherence between different pathways for a transition in a three-level atomic system [14]. However, the experimental condition is district-required. Recently, another pathway to realize an analogous function is called Plasmon-induced transparency (PIT), which rises from the constructive and destructive interference between several resonances with diverse line widths [16]. PIT is recognized as a significant analogy to EIT effects in the metamaterial, which can greatly help us to regulate and control different shapes of metamaterial to achieve the functions needed.

Plenty of nanostructures with different shapes have been proposed to realize filtering functions in recent years [17,18,19]. However, it is more focused on the discussion of the coupling between the bright mode and dark mode [16,17]. Generally, the bright-dark mode coupling can be limited by the resonance of the dark mode. Under these circumstances, the bright mode is strongly coupled to the external radiation, while the dark mode is excited by the bright mode. So the bright and dark modes sometimes interact feebly. This leads to a deficiency of filtering performance. Based on the discussion above, the design of an easy-produced, effective device is in demand.

Graphene is a new-found mono-layer of carbon atoms, which displays many unprecedented properties, like low propagation losses. Recently, graphene-based devices have been investigated extensively in photonics and optoelectronics [20,21,22,23,24], and the application of this new material leads to a number of novel performances. For example, Han et al. [25] and Shi et al. [26] independently developed several pathways to realize PIT effects based on graphene, and a brilliant performance of multiple-channel PIT transparent windows can also be achieved [25]. However, the fabrication and application of graphene-based structures may increase the experimental difficulty.

In this work, in order to approach a better filtering performance with lower practical difficulty in experiments, we propose and demonstrate an easily made metamaterial structure supporting two bright resonant modes. Under the coupling between two bright modes, an obvious transparent peak can be generated. From the simulation results, we can see that the FWHM (the full width at half maximum) of the transparent peak reaches 0.39 THz. Besides, with a little change of the structure, the line width of the transparent window shows a gradual broadening tendency. Furthermore, we introduce the photosensitive silicon into the structure. By adding the pump light to the silicon, the silicon can lead to a variety of resonating characteristics. This causes a dramatic drop of the transmission at 1.32 THz, which indicates a feasible optical switch manipulated by pump beam.

## 2. Materials and Method

**Figure 1:**
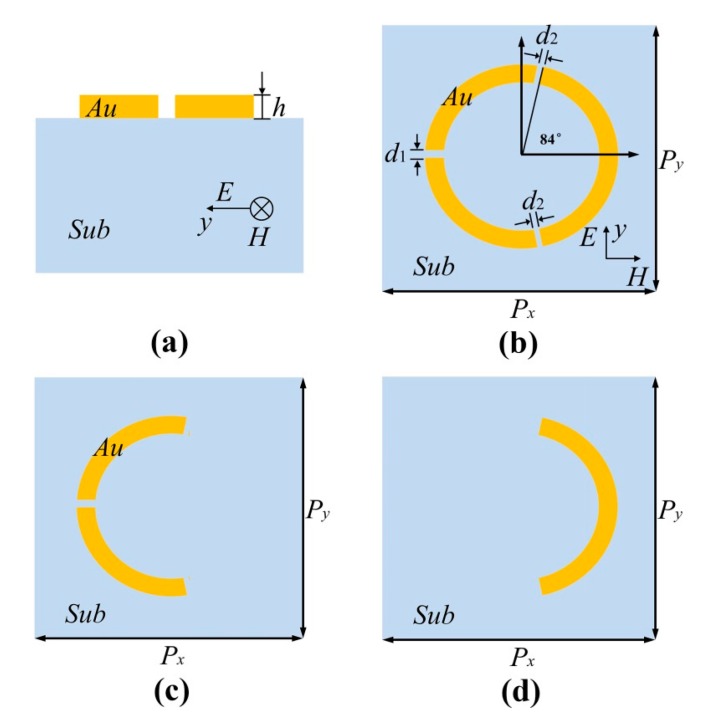
Cross section of the presented metallic three-arc-ring metamaterial (**a**); top view of the metamaterial (**b**); top view of Part A (**c**); top view of part B (**d**).

The schematic diagram of the metamaterial structure of a unit cell is shown in Figure 1a,b. The metamaterial is periodic, but a periodic structure will take too much time on simulation. Hence, we use a single unit cell of the structure with periodic boundary conditions to equal to the periodic structure. The unit cell can be divided into two layers, the metallic (Au) layer on top and dielectric layer beneath. The metallic layer contains three arc rings. The inner radius of the three rings *R*_1_ is 30 μm while the outer radius *R*_2_ is 37 μm. In our coordinate, the angle along the *x*-axis is set as 0°. The two shorter arcs of the structure share the same angular range, which is 89° (88 to 177 and 183 to 272, respectively), while the longer arc ranges from −84° to 84°. The remaining parts between three arc rings are designed as air gaps. The thickness *h*_1_ of the metallic layer is set as 0.2 μm and the conductivity is *σ* = 4.09 × 10^7^ S/m. The thickness *h*_2_ of the substrate is set as 640 μm and the relative refractive index of dielectric substrate is *ε* =1.5. Both metal and substrate are made of non-dispersive materials. The simulating results of the metamaterial are performed by the finite-difference time-domain method, in which the incident radiation is chosen as a plane wave with a *y*-axis polarized electric field, as shown in Figure 1b.

## 3. Simulation Results and Discussion

**Figure 2:**
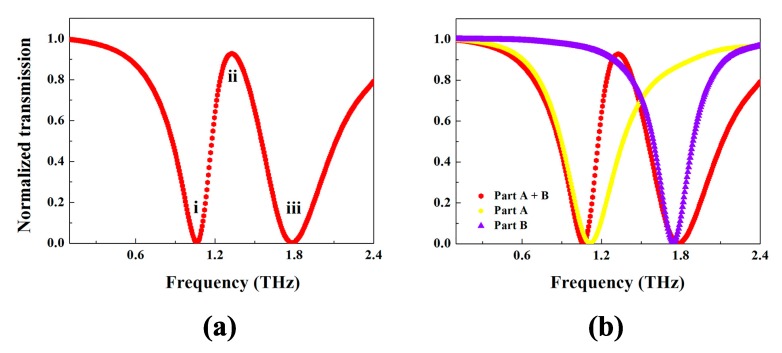
The transmission spectra of the metamaterial (**a**) and the transmission spectrum of the metamaterial, Part A and Part B (**b**).

The red curve in Figure 2a shows the transmission spectra of the metamaterial design depending on the frequency of incident wave. As shown, two resonance dips can be clearly observed. There is a resonance dip at 1.06 THz which is named dip (i) and another resonance dip at 1.78 THz which is called dip (iii). Both dips are approximately non-transparent around each central frequency. Between i and iii, an evident transparent window, which is named peak (ii), is found at the frequency of 1.32 THz, whose transmission can reach 92.75%. The full width at half maximum (FWHM) of the peak comes to 0.39 THz.

To have an insight into the physical picture of the transparent window of the metamaterial structure, two parts of the structure are investigated separately. One is the longer arc-ring on the right side of the structure, and the other consists of the two shorter arc-rings on the left side. For convenience of discussion, we call the longer arc-ring only Part A which is depicted in Figure 1c, and the two shorter arc-rings part B, shown in Figure 1d. The yellow curve of the Figure 2b displays the transmission of part A with central frequency of 1.11 THz, while the blue curve in Figure 2 shows the transmission of part B with its central frequency of 1.72 THz. Both A and B can be excited directly by the incident light, so they act as the so-called bright modes [16]. When two parts join with each other forming the whole structure, a transparent window (ii) appears, as given in the red curve of Figure 2b.

**Figure 3:**
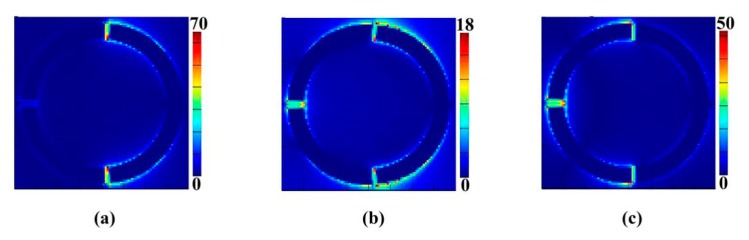
The electric field (|**E**|) distributions for the proposed structure at the resonance frequencies *f*_1_ = 1.06 THz (**a**), *f*_2_ = 1.32 THz (**b**), and *f*_3_ = 1.78 THz (**c**).

The electric field distributions of the whole structure at 1.06, 1.32 and 1.78 THz are respectively attached in Figure 3a–c. As expected, at 1.06 THz, part A dominates the resonance like a dipole resonator. At 1.78 THz, part B resonates as bi-dipole resonator mode that preponderates over part A. When at 1.32 THz, each major bright mode counteracts another one in the overlapping area (at the middle of two resonating frequencies), which weakens the total electric field and finally leads to a higher transmission around 1.32 THz.

## 4. Theory Analyze

Here we discuss the forming process of the transmission. Generally, the transmission T and the absorptivity α of an optical system are decided by the permittivity ε of the system, and *ε* is the function of the incident frequency. Normally, ε simultaneously has a real part and an imaginary part. As ε mainly depends on the effective electric susceptibility$\;\chi_{e}$, T and α can be expressed as below:

(1) $$\alpha \left(\omega \right)=Im\left(\chi_{e}\right)$$

(2) T(ω)=1−α(ω)

To give an insight into the mechanism of the spectra, we should find out how the effective electric susceptibility$\;\chi_{e}$ is influenced by the resonance modes.

We now analyze the effective susceptibility in our metamaterial. As Plasmon-Induced Transparency approximately arises from the coupling effect of two oscillators, we treat both as classical harmonic oscillators analogously. Therefore, the coupled Lorentzian model is applied and the motion function can be derived:

(3) $${\ddot{x}}_{1}\left(t\right)+\gamma_{1}{\dot{x}}_{1}\left(t\right)+\omega_{1}^{2}x_{1}\left(t\right)+\Omega^{2}x_{2}\left(t\right)=\frac{q_{1}E}{m_{1}}$$

(4) $${\ddot{x}}_{2}\left(t\right)+\gamma_{2}{\dot{x}}_{2}\left(t\right)+\omega_{2}^{2}x_{2}\left(t\right)+\Omega^{2}x_{1}\left(t\right)=\frac{q_{2}E}{m_{2}}$$

$x_{1}\left(t\right)=A_{1}e^{i\omega t}$, and $x_{2}\left(t\right)=A_{2}e^{i\omega t}$ are respectively the general solutions of the harmonic oscillator 1 and 2. $A_{1}$, $A_{2}$ represent the amplitude. $\gamma_{1},\omega_{1},q_{1}$ and $m_{1}$ are the damping factor, angular frequency, effective charge, and effective mass of oscillator 1, respectively. For oscillator 2, the expression keeps the same as oscillator 1. Ω is the coupling constant of the structure. We can easily understand each component in the function from the perspective of classical dynamics. The motion of a simple oscillator (like electron) follows the simple harmonic vibration.$\;m\ddot{x}$ is the acceleration of the oscillator and $-m\gamma \dot{x}$ represents the damping force,$-m\omega^{2}x$ is the restoring force. qE on the right side accounts for the effect of electric field force. By solving the function we can get$\;x_{1}\left(t\right)\;$and$\;x_{2}\left(t\right)$. The electric polarization in this case becomes [27]:

(5) $$P\propto q_{1}x_{1}\left(t\right)+q_{2}x_{2}\left(t\right)$$

**Figure 4:**
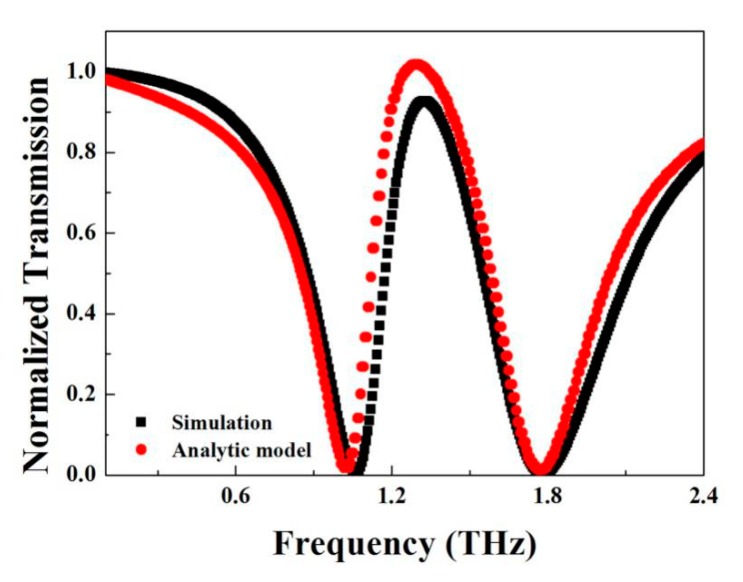
The transmission curve calculated by analytic model (black) as well as the simulated transmission curve (red).

For calculation we define $A=\frac{q_{1}}{q_{2}}$ and $B=\frac{m_{1}}{m_{2}}$ [28] then the expression of effective susceptibility can be derived from (5) as below:

(6) $$\chi_{e}=\frac{K}{A^{2}B}(\frac{A\left(B+1\right)\Omega^{2}+A^{2}\left(\omega^{2}-\omega_{2}^{2}\right)+B\left(\omega^{2}-\omega_{1}^{2}\right)}{\Omega^{4}-\left(\omega^{2}-\omega_{1}^{2}+i\omega \gamma_{1}\right)\left(\omega^{2}-\omega_{2}^{2}+i\omega \gamma_{2}\right)}+i\omega \frac{A^{2}\gamma_{1}+B\gamma_{2}}{\Omega^{4}-\left(\omega^{2}-\omega_{1}^{2}+i\omega \gamma_{1}\right)\left(\omega^{2}-\omega_{2}^{2}+i\omega \gamma_{2}\right)})$$

where $\omega_{1}\;$and $\omega_{2}\;$are the central frequency of oscillator 1 and 2, respectively, $\gamma_{1}$ and $\gamma_{2}$ come from the line-width. Considering the interaction that exists between the two bright modes and oscillator 1 is slightly larger than 2, constant A and B are kept a little more than 1, constant K and coupling constant Ω can be derived from the formula in Refs. [27,28]. Taking all the parameters in (6), the curve of T(ω) can be drawn to estimate the transmissivity. Putting the calculation and simulation results together, we get Figure 4.

**Figure 5:**
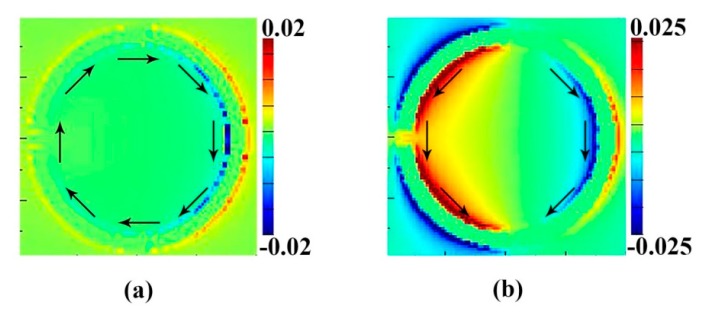
The magnetic field (|**H**_z_|) distributions for the proposed structure at the resonance frequencies of *f*_1_ = 1.06 THz (**a**) and *f*_2_ = 1.78 THz (**b**).

The red curve is the simulation by FDTD (finite-difference time-domain) solutions. The black curve is the theoretical calculation result. The two curves share the same lineshape and common features. The consistency of both curves suggests that Plasmon-Induced Transparency comes from the interaction between two different inter-coupled resonances. The frequency shifts of both resonances from the case of existing alone to staying together can also be described by this interaction as well. This interference between the two modes, in the coupling theory, is principally depicted by the coupling constant Ω. This interaction can also be observed by the magnetic field distribution on z direction Re (H_z_) shown in Figure 5a,b.

We find out that when an incident plane wave reaches the surface, specific field distributions (including E, H and current) are excited and each distribution corresponds with a resonance. From Figure 3 and Figure 5 we can see that the resonances at 1.06 THz and 1.78 THz have different electromagnetic fields. According to Ampere’s law, two resonances support different orientations of current flow. Figure 5a gives the magnetic field distribution |**H**_z_| of the structure resonating around 1.06 THz. A surface current flows clockwise on left arcs. Another strong surface current flows anticlockwise on Part B around 1.78 THz. At 1.32 THz, two opposite current flows destructively interfered with each other, which leads to the weakening effect of the surface current over part B of the structure, which directly gives rise to the transparent phenomena. This is the reason why two resonances may couple with each other and induce a transparent peak at 1.32 THz.

**Figure 6:**
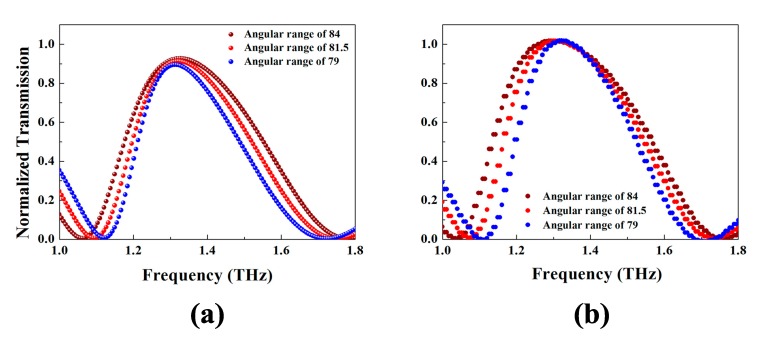
Dependence of the simulated transmission curves on the angular range of Part A (**a**) and the transmission curves calculated by analytic model on the angular range of Part A (**b**).

Next, we discuss the performance of this structure caused by variation of geometric parameter. We simulate the transparent spectra of three cases: The angular range of the longer arc ring is 168°, 163°, and 158° respectively, at the same time fixing the size of the gaps. The transparent spectra of these cases are displayed in Figure 6a. With the angular range declining and the gap fixed, the transparent peak performs a narrowing trend with little change of transmissivity. This is because the central frequency of a dipole oscillator is given by the relation below [29]

(7) $$f\propto \frac{1}{l}$$

of which *l* is the equivalent length of the oscillator. As Part A shortening the central frequency of the single dipole shows a blue shifting tendency. While with the increase of the remaining arcs, the frequency of Part B has a tendency of blue shift. So when they couple with each other, the transparent window narrows. The calculation results by the coupling model in these circumstances are depicted in Figure 6b. The narrowing inclination offers us a channel to limit the frequency range of the transparent window.

## 5. Potential Applications

**Figure 7:**
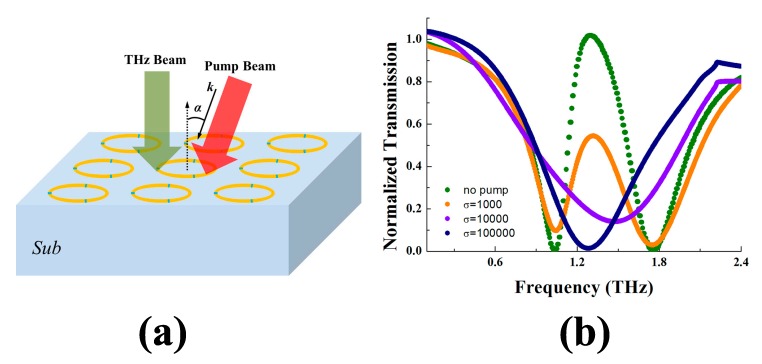
The structure with pump beam illuminated (**a**) and the transmission spectra of the proposed structure under no pump beam (olive), σ = 1000 (orange), σ = 10,000 (violet) and σ = 100,000 (navy) (**b**).

In this section, we discuss the potential functional applications as a device. Plenty of efforts around pump-beam sensitive medium in metamaterial [30,31,32,33] have been proved to realize extensive performance controls and functional regulations. In this section, we introduce the photosensitive silicon into the structure to study the switching function. Photosensitive silicon is a kind of semiconductor whose conductivity σ can be manipulated by light. When the silicon is illuminated by optical radiation of proper frequency, an excess carrier density can be generated [30]. This injection of carriers may greatly elevate the conductivity of the silicon. To realize a switching performance, we fill the silicon into the gap and choose the near-infrared laser beam with a central wavelength of 800 nm to excite the excess carriers and change the conductivity of the silicon gap [33]. The direction has an angle α = 20° with the normal direction of the metamaterial, as shown in Figure 7a, and the pump laser beam is able to cover the surface of the metamaterial. The conductivity corresponds to the laser intensity; hence we can adjust the σ by controlling the laser [31,32,33]. Here we set three different σ to study the change in the transmission curve. The simulation results are given in Figure 7b. The olive curve is the transparent spectra with no pump beam. The orange, violet and navy curves are the transparent spectra when σ = 1000, 10,000 and 100,000, respectively. At 1.32 THz, without pumping, the structure is nearly transparent. However, with the conductivity of the silicon dramatically increased, the structure starts to block the light. When σ reaches 1 × 10^5^ or larger, the structure strongly forbids the transition of the incident wave of 1.32 THz. The variety from region pass to region stop permits an application of light switching.

## 6. Conclusions

In conclusion, we design a three-arc-ring artificial terahertz metallic metamaterial, which has a mirror symmetric character and is easy to fabricate. The structure shows an obvious Plasmon-Induced Transparency at (ii) and strongly blocks the light at (i) and (iii). The relative maximum transmission can reach 93%. The coupled Lorentzian oscillator model is utilized to analyze the coupling interaction between two bright modes. It is found that the simulation and theoretical calculation matches well. The cause of the transparent peak is the coupling effect between two modes. Moreover, a narrowing trend of the transparent peak is discussed when the angular range of the longer arc-ring decreases. Finally, we proposed a possible functional utilization of this metamaterial. With the use of photoconductive silicon, a frequency-dependent tunable on-off can be realized.
